# Extended indirect calorimetry with isotopic CO_2_ sensors for prolonged and continuous quantification of exogenous *vs*. total substrate oxidation in mice

**DOI:** 10.1038/s41598-019-47977-w

**Published:** 2019-08-08

**Authors:** José M. S. Fernández-Calleja, Lianne M. S. Bouwman, Hans J. M. Swarts, Annemarie Oosting, Jaap Keijer, Evert M. van Schothorst

**Affiliations:** 1 Human and Animal Physiology, Wageningen University, De Elst 1, Wageningen, 6708 WD The Netherlands; 2 Danone Nutricia Research, Uppsalalaan 12, Utrecht, 3584 CT The Netherlands

**Keywords:** Mouse, Fat metabolism

## Abstract

Indirect calorimetry (InCa) estimates whole-body energy expenditure and total substrate oxidation based on O_2_ consumption and CO_2_ production, but does not allow for the quantification of oxidation of exogenous substrates with time. To achieve this, we incorporated ^13^CO_2_ and ^12^CO_2_ gas sensors into a commercial InCa system and aimed to demonstrate their performance and added value. As a performance indicator, we showed the discriminative oscillations in ^13^CO_2_ enrichment associated with food intake in mice fed diets containing naturally low (wheat) *vs* high (maize) ^13^C enrichment. To demonstrate the physiological value, we quantified exogenous *vs* total carbohydrate and fat oxidation continuously, in real time in mice varying in fat mass. Diet-induced obese mice were fed a single liquid mixed meal containing ^13^C-isotopic tracers of glucose or palmitate. Over 13 h, ~70% glucose and ~48% palmitate ingested were oxidised. Exogenous palmitate oxidation depended on body fat mass, which was not the case for exogenous glucose oxidation. We conclude that extending an InCa system with ^13^CO_2_ and ^12^CO_2_ sensors provides an accessible and powerful technique for real-time continuous quantification of exogenous and whole-body substrate oxidation in mouse models of human metabolic physiology.

## Introduction

Indirect calorimetry (InCa) has been essential to understand human whole-body energy metabolism and fuel selection for more than a century^1^. It allows for the estimation of energy expenditure (EE) and of substrate utilisation by calculation of the respiratory exchange ratio (RER) from O_2_ consumption and CO_2_ production measurements. In the mouse, indirect calorimetry is the preferred technique to measure energy expenditure continuously^2^. However, estimations of RER represent only the whole-body net balance of substrates oxidised and do not distinguish between endogenous and exogenous (dietary) metabolic substrates. This knowledge can be obtained with the use of metabolic tracers, which are compounds that behave identically to the compound of interest, but can be analytically distinguished from them, for instance by mass difference.

The stable natural isotope ^13^C has been widely used in metabolic studies for the past five decades^3^. Next to ^12^C, ^13^C is the second most abundant isotope of elemental carbon, representing about 1% of total terrestrial carbon^4^. Based on their distinct CO_2_ fixation mechanisms, plants classified as C4 (*e.g*. maize or corn, and sugar cane) have a naturally higher ^13^C abundance compared to C3 plants (*e.g*. wheat and sugar beet), and ingredients derived from C4 plants are often used as natural metabolic tracers^5^.

Upon oxidation in the body, ^13^C atoms in metabolic substrates are excreted as ^13^CO_2_ which can be measured in breath samples and is usually given as atom % ^13^CO_2_ enrichment^3^. While this provides a qualitative measurement of exogenous substrate oxidation, quantitative evaluation of substrate oxidation rates provides better insight into metabolic physiology and allows direct comparison between exogenous and endogenous substrates. Such quantitative measurements require knowledge of CO_2_ production rates, and this has been regularly determined in human studies by InCa^3^. Only recently, livestock animal nutritionists and comparative biologists have also started to combine ^13^CO_2_ enrichment analysis with InCa to calculate substrate oxidation rates^5–8^. Although informative, metabolic tracer research in animal models, like the mouse, rarely includes measurements of CO_2_ production rates and is limited by the number of breath samples that can be collected and analysed for ^13^CO_2_ enrichment^9–13^. Continuous quantification of total CO_2_ production together with ^13^CO_2_ enrichment would allow precise measurement of responses to metabolic tracers in models of human physiology. To our knowledge, there have been only two attempts to obtain quantitative measurements of substrate oxidation in mice using ^13^CO_2_ enrichment data in tandem with InCa over prolonged study times, and they involved expensive equipment^14^ or labour-intensive sampling^15^.

We have recently shown that a commercial InCa system can be successfully extended to incorporate analysis of other gases^16^, in this case measurement of the gut microbiota fermentation gases. The inclusion of hydrogen (H_2_) and methane (CH_4_) sensors resulted in a system that offers a tool for more detailed phenotyping than conventional InCa for nutritional interventions in mice. Here, we have also incorporated ^13^CO_2_ and ^12^CO_2_ sensors into the same commercial InCa system and demonstrate their usefulness technically and physiologically. The system was able to characterise changes in natural ^13^CO_2_ enrichment based on 24 h feeding cycles and the consumption of diets with distinct ^13^C signatures. Moreover, real-time ^13^CO_2_ enrichment measurements linked to conventional InCa added value to current established metabolic phenotyping methodologies such as refeeding challenge tests, by not only quantifying exogenous and endogenous oxidation rates, but also quantifying the oxidative disposal of glucose and fat ingested with a meal in the context of diet-induced obesity.

## Results

### Integration of ^13^CO_2_ and ^12^CO_2_ sensors into the InCa system and analysis of natural dietary ^13^C enrichment

**Figure 1:**
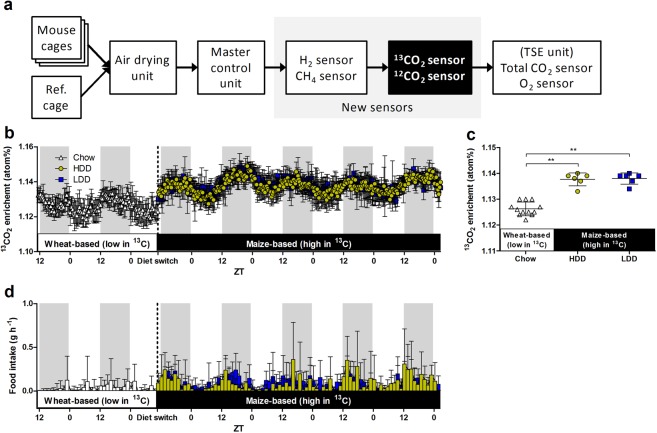
Extended InCa system with ^13^CO_2_ and ^12^CO_2_ gas analysers and technical validation with mice (first mouse study). (**a**) Simplified configuration of the system showing elements connected in parallel (12 mouse cages and one reference cage) and in in series (six gas sensors). Arrows indicate the direction of the airflow. (**b**–**d**) Female mice raised on wheat-based chow (*n* = 12) were acclimatised to the InCa system and then switched to one of two diets containing 57% w/w maize starch (HDD and LDD, both containing the same proportion of starch; *n* = 6 per diet). (**b**) ^13^CO_2_ enrichment calculated from ^13^CO_2_ and ^12^CO_2_ concentrations were measured continuously for 6.5 d. (**c**) Mean ^13^CO_2_ enrichment over the complete periods on wheat-based chow or maize-based diets. (**d**) Food intake was measured before and during HDD and LDD feeding. Shaded areas represent the dark phase. **P ≤ 0.01. Data is presented as mean ± SD. InCa, indirect calorimetry; Ref., reference cage; ZT, Zeitgeber time.

We first evaluated the overall performance of the newly integrated ^12^CO_2_ and ^13^CO_2_ sensors into the InCa system (Fig. 1a) with a 5 d test with empty cages to measure diurnal fluctuations in ambient air. Median ambient levels during this test were 5.30 ppm ^13^CO_2_ (4.42, 6.08; range) and 462.8 ppm ^12^CO_2_ (449.7, 535.2; range), respectively. This corresponded to a median of 1.130 atom% ^13^CO_2_ (0.960, 1.159; range).

After these initial tests, female mice raised on wheat-based chow (a C3 plant, thus low in ^13^C) were placed into the InCa system and ^13^CO_2_, ^12^CO_2_, and other gas concentrations were recorded continuously for 48 h (Fig. 1b). Once animals were acclimatised, the wheat-based chow was exchanged for one of two maize-based semi-purified diets, a highly digestible-starch diet (HDD) and a lowly digestible-starch diet (LDD), each containing the same amount of maize starch (a C4 plant, thus naturally high in ^13^C). Oscillations in ^13^CO_2_ enrichment followed a circadian pattern, reaching the lowest levels during the light phase (LP) and highest levels during the dark phase (DP; Fig. 1b); this pattern was similar to the food intake (FI) pattern (Fig. 1d). Upon the switch to a maize-based diet, overall ^13^CO_2_ enrichment levels were significantly higher (*P* = 0.0002), independent of type of maize starch used, with a mean difference of 0.011 and 0.012 atom% for HDD and LDD, respectively, compared to chow (Fig. 1c). The ^13^C enrichment of the diets measured by elemental analysis isotope ratio mass spectrometry (EA-IRMS) was 1.078 atom% for chow and 1.085 atom% for both HDD and LDD, thus a natural enrichment difference of 0.008 atom%, which is close to the observed ^13^CO_2_ differences in the expired air. Additionally, analysis of a separate group of mice that remained on chow instead of switching to HDD or LDD confirmed that the increase in mean ^13^CO_2_ enrichment was driven mainly by dietary ^13^C enrichment and less so by increased FI (Supplementary Fig. S1). Finally, to assess the functionality of the newly incorporated sensors as part of conventional respirometry, we recalculated EE by substituting total VCO_2_ values obtained with a single sensor with the sum of V^13^CO_2_ and V^12^CO_2_ values obtained from two sensors. Recalculated and original 24 h EE values were almost identical (41.03 ± 0.78 *vs* 41.03 ± 0.77 kJ d^–1^, respectively, Student’s *t*-test *P* = 0.994; linear regression, Y = 0.9993X + 0.0002; r^2^ = 0.999).

### Refeeding metabolic response in diet-induced obesity

**Figure 2:**
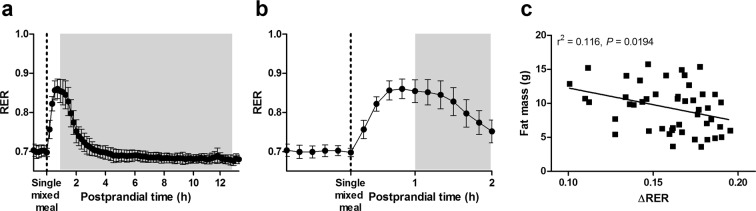
Refeeding metabolic response to a liquid mixed meal and its relation to body fat mass (FM) in mice fed a HFD for nine weeks (second mouse study). (**a**) Respiratory exchange ratio (RER) was measured in PW 14–15 after gavage of a single liquid mixed meal containing 36 energy% glucose and 64 energy% fat (*n* = 47). Data is obtained from conventional indirect calorimetry measurements (VO_2_ and VCO_2_). Data is presented as mean ± SD. Shaded areas represent the dark phase. (**b**) Data from panel (**a**), emphasizing the early postprandial 2 h period. (**c**) Refeeding metabolic response (ΔRER), measured as the increase from baseline RER to RER at 44 min postprandial, correlates negatively with FM. PW, postnatal week.

After 9 weeks on a high-fat-diet (HFD), mice that were previously fed LDD *vs* HDD for 3 weeks had a slightly higher fat mass (FM) and gained more FM over the period on HFD, however, BW was not significantly different between the groups (Supplementary Table S1). The total group of 48 mice on HFD had a FM ranging from 5.52 g up to 15.88 g (288%; Table 1) and was metabolically fairly homogeneous, regardless of prior LDD or HDD feeding (Supplementary Table S1 and Supplementary Fig. S2). The metabolic response to refeeding was tested using ^13^C-labelled liquid mixed meals, and the data derived from the ^13^CO_2_ and ^12^CO_2_ sensors allowed us to detect exogenous tracer (^13^C) as a marker for the total specific substrate, simultaneously with overall substrate oxidation based on O_2_ consumption and total CO_2_ production. Baseline RER values before administration of the liquid mixed meal were 0.70 ± 0.01 (mean ± SD; Fig. 2). The median peak RER (glucose oxidation) was achieved after 44 min (22, 88; range) upon feeding, and values declined thereafter until full fat oxidation^17^ was reached again after approximately 140 min. The refeeding RER response, defined as the increase from baseline RER to RER at 44 min (∆RER, Table 1), was negatively correlated to FM (measured directly prior to InCa measurements; Fig. 2c). In line, also BW (Spearman r = –0.386, *P* = 0.0074; *n* = 47) and LM (r^2^ = 0.245, *P* = 0.0004; *n* = 47) were negatively correlated to ∆RER. Serum insulin levels 45 min post-prandially were also negatively correlated to ∆RER in a subgroup of animals that were fasted and challenged again with the liquid mixed meal before sacrifice (r^2^ = 0.421, *P* = 0.0011; *n* = 22).

**Table 1:** Metabolic characteristics of the mice after HFD-feeding for nine weeks (second mouse study).

Parameter	Outcome
BW (g)^a^	31.57 (24.82, 38.22)
FM (g)^a^	10.21 (5.52, 15.88)
LM (g)^a^	19.89 (17.68, 21.64)
FM gain (g)^b^	8.66 (4.41, 14.06)
FI (g)^b^	188.38 (161.67, 226.33)
24 h EE (kJ d^–1^)^c^	44.24 (38.66, 51.45)
24 h RER^c^	0.81 (0.74, 0.89)
∆RER (RER_44_ – RER_0_)^c^	0.16 (0.10, 0.20)
Fasting glucose (mmol l^–1^)^a^	6.0 (4.9, 7.2)
Fasting insulin (ng ml^–1^)^a^	2.65 (0.85, 8.95)
Postprandial glucose (mmol l^–1^)^a^	6.9 (3.3, 9.1)
Postprandial insulin (ng ml^–1^)^a^	4.40 (1.47, 9.22)

^a^Measured in PW 15 (end of HFD). ^b^Measured from PW 7 (start of HFD) to PW 15. ^c^Measured in PW 14–15. HFD, high-fat diet; BW, body weight; FM, fat mass; LM, lean mass; FI, food intake; EE, energy expenditure; RER, respiratory exchange ratio (mean of 24 h). Data is presented as means and range, *n* = 48 (except ∆RER, *n* = 47; fasting glucose and insulin *n* = 24; and postprandial glucose and insulin, *n* = 23).

### Oxidation of exogenous (tracer) glucose and fat

**Figure 3:**
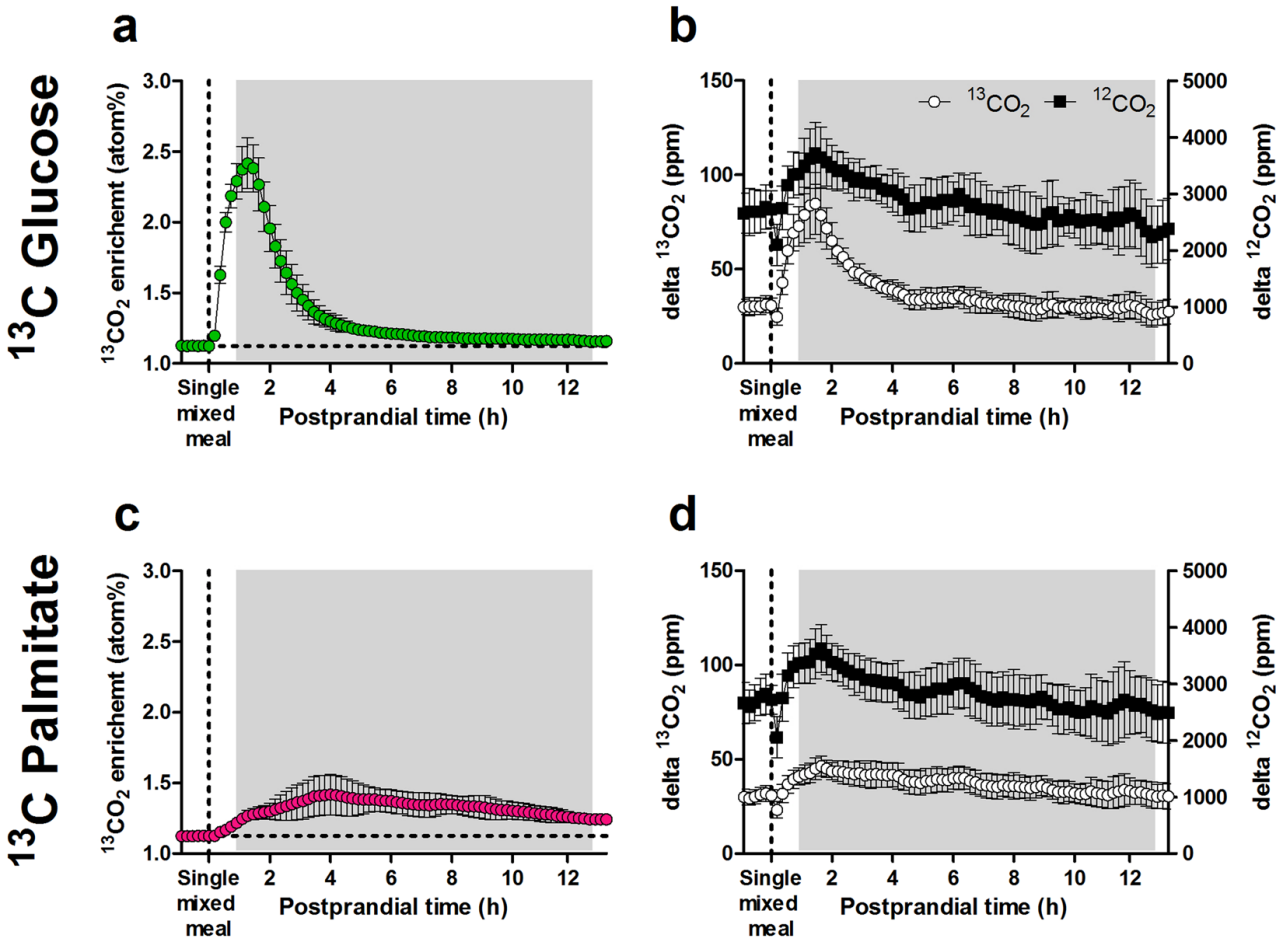
Kinetics of instantaneous ^13^CO_2_ enrichment measured by extended InCa after gavage of a single liquid mixed meal containing ^13^C-labelled tracers, in mice fed a HFD for nine weeks (second mouse study). (**a**) ^13^CO_2_ enrichment after ingestion of the ^13^C glucose liquid mixed meal followed for 13 h (*n* = 23), calculated from ^13^CO_2_ and ^12^CO_2_ concentrations. (**b**) ^13^CO_2_ (left y-axis) and ^12^CO_2_ (right y-axis) concentrations used to calculate ^13^CO_2_ enrichments as shown in panel (**a**), expressed as the difference (delta) from gas concentrations measured in mouse-occupied cages minus gas concentrations in the reference cage (*i.e*. gas production). (**c**) ^13^CO_2_ enrichment after ingestion of the ^13^C palmitate liquid mixed meal (*n* = 24). (**d**) ^13^CO_2_ (left y-axis) and ^12^CO_2_ (right y-axis) concentrations used to calculate ^13^CO_2_ enrichments as shown in panel (**c**). The horizontal dotted lines indicate baseline ^13^CO_2_ enrichment. Shaded areas represent the dark phase. Data is presented as mean ± SD. PW, postnatal week.

The liquid mixed meal was enriched with metabolic ^13^C tracers for glucose or palmitate to investigate dietary fuel partitioning between storage and oxidation, continuously and in real time. Thus, all animals received a liquid mixed meal of identical macronutrient composition, but containing either ^13^C glucose or ^13^C palmitate, and exhaled ^13^CO_2_ was measured to reflect the oxidation of tracer and total glucose and palmitate contained in the meal. Concentrations of ^13^CO_2_ and ^12^CO_2_ were first computed as ^13^CO_2_ enrichment to characterise exogenous fuel oxidation qualitatively (Fig. 3). The label in the ^13^C glucose liquid mixed meal appeared in breath quickly in a single peak at 79 ± 12 min (mean ± SD), and ^13^CO_2_ enrichment fell almost back to baseline by the end of the 13 h post-meal period (final enrichment 1.159 ± 0.012 atom%, mean ± SD; Fig. 3a). In contrast, the label in the ^13^C palmitate liquid mixed meal appeared later and at a slower rate, generally peaking around 4 h (with individual animals showing multiple peaks), and continued to appear by the end of the 13 h period (final enrichment 1.242 ± 0.038 atom%, mean ± SD; Fig. 3c). The underlying concentrations of ^13^CO_2_ and ^12^CO_2_ used to calculate ^13^CO_2_ enrichments are shown in Fig. 3b,d.

**Figure 4:**
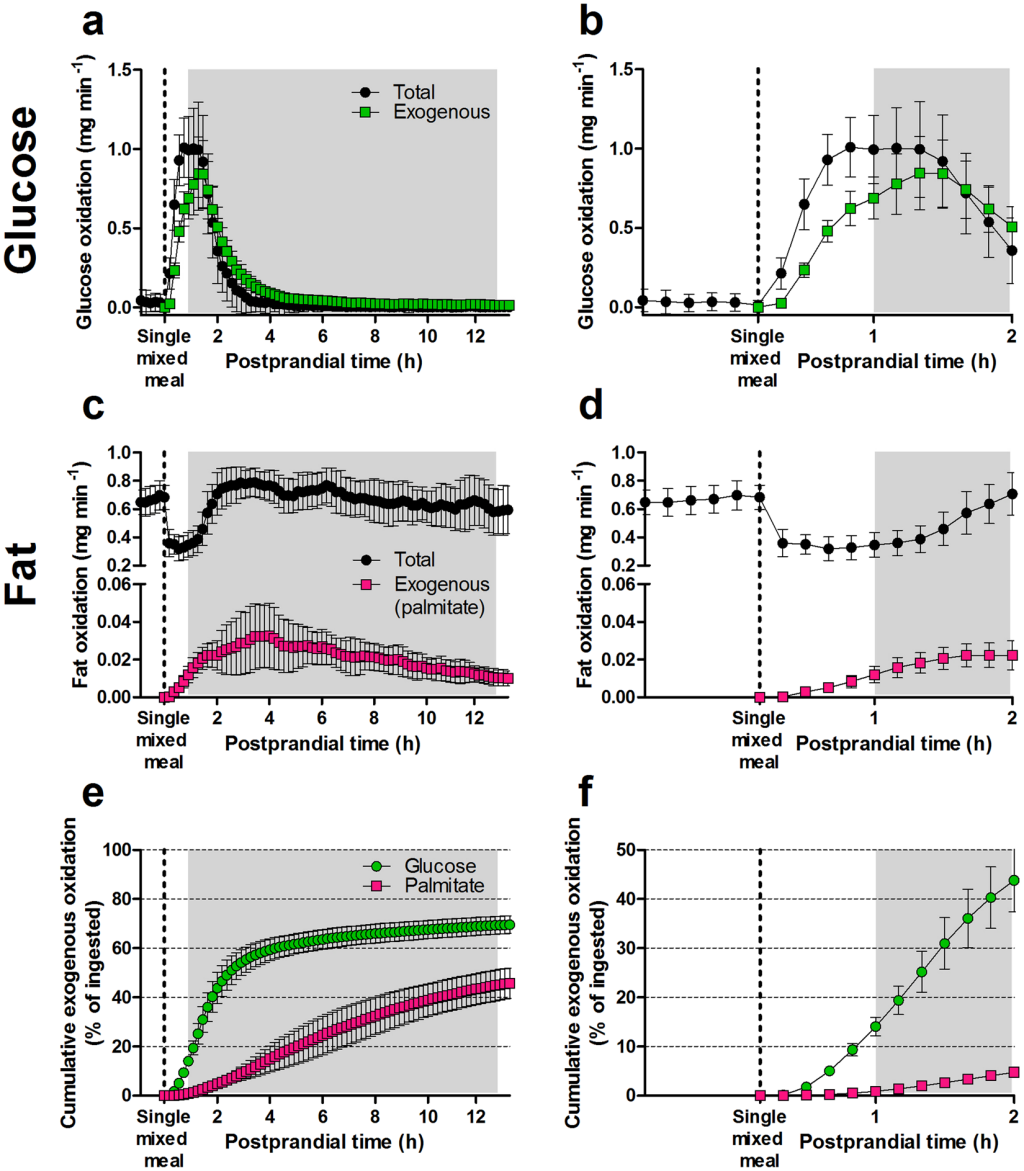
Kinetics of oxidation of total and exogenous metabolic substrates after gavage of a labelled liquid mixed meal, in mice fed a HFD for nine weeks (second mouse study). (**a**) Instantaneous total (*n* = 47) and exogenous (*n* = 23) glucose oxidation after administration of the ^13^C glucose liquid mixed meal until the end of the measurements. (**b**) Data from panel (**a**), emphasizing the early 2 h postprandial period. (**c**) Instantaneous total fat oxidation (*n* = 47) and exogenous palmitate oxidation (*n* = 24) after administration of the ^13^C palmitate liquid mixed meal. (**d**) Data from panel (c), emphasizing the early 2 h postprandial period. (**e**) Cumulative exogenous glucose (*n* = 23) and palmitate oxidation (*n* = 24) after administration of the labelled liquid mixed meals until the end of the measurements. (**f**) Data from panel (e), emphasizing the early 2 h postprandial period. Exogenous substrate oxidation data is calculated from conventional InCa data (VCO_2_) and ^13^CO_2_ and ^12^CO_2_ data. Shaded areas represent the dark phase. Data is presented as mean ± SD. InCa, indirect calorimetry; PW, postnatal week.

To quantify and dissect exogenous substrate oxidation rates, ^13^CO_2_ enrichment data was combined with VCO_2_ production rates and compared to total substrate oxidation, *i.e*. endogenous and exogenous, obtained from conventional InCa equations (Fig. 4). Since the substrate oxidation kinetics of HDD and LDD mice were almost identical (Supplementary Fig. S2), the data was further analysed and plotted as one group. Exogenous glucose oxidation (EGO) and total glucose oxidation (TGO) rates followed in general similar kinetics (Fig. 4a,b). However, EGO achieved a later peak compared to TGO, with a median peak at 77 min (44, 132; range; *n* = 23) and 66 min (33, 99; range; *n* = 47), respectively (Mann Whitney, *P* = 0.0002; Fig. 4a,b). Maximal EGO and TGO were 0.934 ± 0.212 and 1.211 ± 0.213 mg min^–1^ (mean ± SD), respectively. Thereafter EGO and TGO started to decline until they became negligible, with EGO generally remaining slightly higher than TGO (Fig. 4a), likely due to underestimation of total glucose oxidation (see Discussion). In contrast to glucose oxidation, exogenous fat oxidation (EFO), accounting only for palmitic acid, and total fat oxidation (TFO) rates showed clearly different kinetics (Fig. 4c,d). Immediately after administration of the liquid mixed meal, EFO started to rise while TFO was abruptly suppressed (Fig. 4c,d). Later TFO returned back to baseline around 2 h and EFO peaked around 4 h postprandial (Fig. 4c,d). Maximal EFO and TFO were 0.047 ± 0.013 and 0.936 ± 0.069 mg min^–1^ (mean ± SD), respectively. Both EFO and TFO tended to decrease towards the end of the measurements at 13 h but remained above the baseline (Fig. 4c).

Cumulatively, EGO was 111.7 mg (97.5, 122.8; median and range) and TGO 103.5 mg (68.5, 159; median and range) during the complete post-meal measurement period, from consumption of the liquid mixed meal 1 h before the DP and until the end of the DP (13 h in total). This amount of exogenous glucose oxidised corresponded to 69.6% (61.3, 76.6; median and range) of the total dose administered with the liquid mixed meal (Fig. 4e,f). In comparison, EFO was 16.4 mg (9.6, 18.7; median and range) and TFO 511.0 mg (404.2, 635.4; median and range) over the whole period. For exogenous palmitate oxidised, this amount corresponded to 47.6% (27.7, 52.6; median and range) of the oral dose (Fig. 4e,f). The percentage of dose oxidised was more variable for exogenous palmitate (CV = 13.4%) than for exogenous glucose (CV = 5.1%).

### Exogenous substrate oxidised in relation to body composition

We then attempted to explain the variation in exogenous fuel selection in diet-induced obese mice by correlating cumulative oxidation data with FM (determined directly before InCa). TFO was positively correlated to FM (0–2 h: r^2^ = 0.438, *P* < 0.0001; 2–13 h: r^2^ = 0.467, *P* < 0.0001; *n* = 47), while EGO, TGO, and EFO did not reach a statistically significant correlation, neither during the early postprandial period from 0–2 h, nor from 2–13 h after ingestion of the liquid mixed meal.

**Figure 5:**
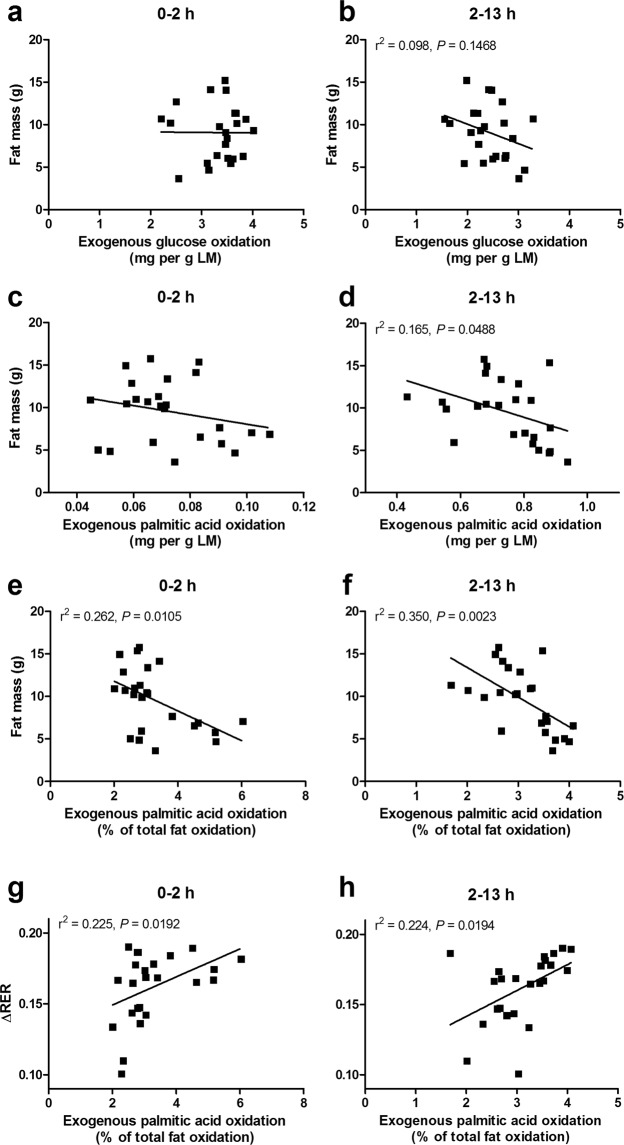
Correlation analysis between cumulative exogenous glucose or fat oxidation after gavage of a labelled liquid mixed meal and body fat mass and ∆RER, in mice fed a HFD for nine weeks (second mouse study). Exogenous glucose oxidation from 0–2 h (**a**) or 2–13 h postprandial (**b**) expressed per unit of LM is not significantly correlated to FM. Exogenous palmitic acid oxidation from 0–2 h postprandial expressed per unit of LM is not significantly correlated to FM (**c**), but it is negatively correlated to FM 2–13 h after ingestion of the liquid mixed meal (**d**). Exogenous palmitic acid oxidation expressed as a percentage of total fat oxidation correlates negatively with FM, both from 0–2 h postprandial (**e**) and from 2–13 h after ingestion of the liquid mixed meal (**f**). Exogenous palmitic acid oxidation relative to total fat oxidation correlates positively with ∆RER, both from 0–2 h postprandial (**g**) and from 2–13 h after ingestion of the liquid mixed meal (**h**). *n* = 24. PW, postnatal week.

Although LM showed a narrow range of values (17.68, 21.64; Table 1), it correlated positively with FM (r^2^ = 0.349, *P* < 0.0001; *n* = 48), and thus differences in total oxidative tissue may explain the correlations of substrate oxidation with FM. Therefore, oxidation data were also expressed relative to LM. Correlations of EGO with FM remained non-significant (Fig. 5a,b), and TGO correlated negatively with FM only during the early postprandial period (0–2 h: r^2^ = 0.101, *P* = 0.0296; 2–13 h: r^2^ = 0.039, *P* = 0.1861; *n* = 47). Positive correlations of TFO with FM (0–2 h: r^2^ = 0.355, *P* < 0.0001; 2–13 h: r^2^ = 0.2838, *P* = 0.0001; *n* = 47) remained significant. Moreover, after accounting for LM, EFO correlated negatively with FM (2–13 h: r^2^ = 0.165, *P* = 0.0488; *n* = 24), only during the late postprandial and post-absorptive period (Fig. 5c,d).

Expressed relative to TFO, EFO was negatively correlated to FM (0–2 h: r^2^ = 0.262, *P* = 0.0105; 2–13 h: r^2^ = 0.350, *P* = 0.0023; *n* = 24; Fig. 5e,f). Additionally, EFO relative to TFO was positively correlated with ∆RER (0–2 h r^2^ = 0.225, *P* = 0.0192; 2–13 h: r^2^ = 0.224, *P* = 0.0194; *n* = 24; Fig. 5g,h), an unsuspected outcome since the refeeding response is generally qualified by a switch to glucose oxidation and concomitant suppression of fat oxidation.

In metabolic tracer studies with humans, particularly when using isotopically labelled fatty acids, it is important to account for label sequestration in the body bicarbonate pool and in exchange reactions in the TCA cycle, and this can be achieved by applying an acetate correction factor to the oxidation calculations^18^. When the labelled fatty acids are ingested, this factor is called the dietary acetate recovery factor (dARF) and its value depends on whole-body adiposity and fasting insulin levels^19^. To our knowledge, a dARF is yet to be determined for mice. Since ^13^C label sequestration from palmitate could also depend on FM in mice and lead to potentially wrong interpretations of exogenous oxidation data, we tested the potential consequences of applying this factor. In view of a lack of dARF in mice, we applied the dARF known for normal-weight and obese humans (13.3, 31.3 kg FM; range) to individual mice, assuming a linear relationship between FM and the dARF. Including a dARF in the calculations of EFO led to much higher label recoveries, with a median of 99.0% (59.2, 116.0; range) exogenous palmitate oxidation of dose administered. After accounting for label sequestration in this way, EFO relative to LM was no longer significantly correlated to FM. However, EFO relative to TFO remained negatively correlated to FM (0–2 h: r^2^ = 0.188, *P* = 0.0345; 2–13 h: r^2^ = 0.224, *P* = 0.0194; *n* = 24), and also remained positively correlated to ∆RER (0–2 h: r^2^ = 0.211, *P* = 0.0241; 2–13 h: r^2^ = 0.195, *P* = 0.0306; *n* = 24).

## Discussion

We have incorporated two new gas sensors, for ^13^CO_2_ and ^12^CO_2_, into a commercial indirect calorimetry (InCa) system for mice. Using this extended InCa system, we were able to detect long-term continuous changes in ^13^CO_2_ enrichment based on the natural ^13^C content of the diet (wheat-based *vs* maize-based diets) and to analyse and dissect the use of exogenous or endogenous body fuels (feeding *vs* fasting conditions). Furthermore, the extended system was particularly suitable for real-time and continuous quantification of exogenous substrate oxidation using ^13^C-labelled ingredients. Combined with conventional InCa, providing mice a liquid mixed tracer meal revealed that with increased FM a blunted switch to glucose oxidation was accompanied with a decreased utilisation of exogenous dietary palmitate. Thus, extended InCa with ^13^CO_2_ and ^12^CO_2_ sensors provided a deeper understanding of metabolic phenotypes.

Quantifying the oxidative disposal of exogenous metabolic substrates has been used in many human metabolic studies^3^. However in mice, to the best of our knowledge, only one published study quantified oxidation of glucose and fat ingested in a meal using stable isotopic tracers^14^. Due to the choice of different tracers (^13^C glucose replacing sucrose, and ^13^C trioleate replacing soybean oil) and the lack of data on dose recovery percentages^14^, it is not possible to compare our conclusions regarding quantification of oxidised substrates. Comparison with other species and protocols is not easy, given the diversity in metabolic rates, doses, tracers, study durations, and other experimental conditions. Nevertheless, our results are consistent with the general notion that ingested fats are less readily oxidised than carbohydrates^5^, and with the observation that maximal oxidation rates of ingested palmitate were one order of magnitude smaller than for glucose, as was investigated in another rodent, *Phodopus sungorus*^8^. At the same time, the cumulative oxidation of ingested palmitate was highly variable compared to that of ingested glucose (CV = 13.4% *vs* 5.1% for palmitate and glucose, respectively). Together, these data reinforce the concept that glucose balance is more tightly controlled than fat balance, at least in the short-term^20,21^. Arguably, glucose oxidation is the most straightforward postprandial oxidation fuel strategy, as fat oxidation has been shown to depend on chain length and degree of saturation^22–25^ and even physical structure^11,26^.

Using ^13^CO_2_ and ^12^CO_2_ sensors in combination with InCa allows for a more refined interpretation of the capacity to adjust substrate oxidation to its availability, which is also referred to as metabolic flexibility^27^. We^28–32^ and others^9,33^ have used this concept as a strategy for metabolic phenotyping in mice by measuring the RER. However, it is unclear to what extent this adaptation to available substrates involves the oxidation of exogenous substrates *per se*, since this question cannot be answered calculating RER alone. As could be expected as part of an obese metabolic phenotype, a blunted RER response to refeeding was correlated to increased body fat mass in our mice (Fig. 2c). Remarkably, this was only reflected in the total amount of glucose oxidised in the early postprandial period, while the total amount of ingested glucose that became oxidised was not related to fat mass. This could suggest that hepatic uptake and regulation of ingested glucose remained unaffected, while meal-induced glucose oxidation may have been impaired at the systemic level by competition with circulating fatty acids in obese mice.

The ability to study exogenous fat oxidation with isotopic sensor extended InCa provided renewed insights to interpret the fasting-and-refeeding challenge as a test of metabolic flexibility. Remarkably, exogenous palmitate was oxidised directly after administration of the meal, a time when fat oxidation was suppressed at the whole body level attributable to increased circulating insulin levels^34^. Likely, intestinal enterocytes were the first site of oxidation of exogenous palmitate directly after consumption of the meal, which is supported by human data showing that orally administered trioleate is oxidised more readily than intravenous trioleate^24^, and that intestinal mucosa can readily oxidise about 17% of luminal palmitate in rats^35^. In line, fish omega-3 fatty acids *vs* plant omega-3 fatty acids were shown to induce fatty acid oxidation rates in the small intestines of mice^36^. Similar to what can be derived from RER data, our findings seem to indicate that oxidation of exogenous fatty acids is not a feature of metabolic flexibility in the early postprandial period. However, we found that exogenous palmitic acid oxidation correlated negatively with body fat mass in the late postprandial and post-absorptive periods (defined in this study as 2–13 h after ingestion of the liquid mixed meal; Fig. 5d). Moreover, the proportion of exogenous palmitic acid oxidised relative to total fatty acid oxidation not only correlated negatively with fat mass (Fig. 5e,f), but also correlated positively with ∆RER (Fig. 5g,h). Although speculative, it is possible that a blunted exogenous fatty acid oxidation is an unrecognised feature of metabolic inflexibility in response to a mixed meal.

Our observed ambient ^13^CO_2_ enrichment levels (1.130 atom%) were not completely consistent with literature values, which are reported to vary between 1.100 to 1.103 atom% (refs.^3,37,38^). This may be due to the ventilation characteristics of the animal room, because the magnitude of the difference in ^13^CO_2_ enrichment between wheat- and maize-based diets (0.012 atom%) was close to the measured values of the diets using EA-IRMS (0.008 atom%). Moreover, the circadian variation in exhaled ^13^CO_2_ enrichment of mice on non-labelled diets was as anticipated^5,39^. We also observed during the late postprandial and post-absorptive period following the ^13^C-labelled meals, that exogenous glucose oxidation exceeded total glucose. This likely is a direct consequence of the fact that a large part of the RER values fell below 0.704. Below this value, net glucose utilisation is considered to be zero (Péronnet & Massicotte)^17^. More precisely, glucose oxidation can continue to take place, with ketogenesis and gluconeogenesis from amino acids contributing to a decrease in RER and effectively leading to an underestimation of glucose oxidation^1,17^. In our study, such processes could be expected as the mice were fasted for 24 h prior to the food restriction, which was with a high fat (*i.e*. a relative ketogenic) diet and returned to short-term negative energy balance already 4 h after ingesting the single mixed meal. The higher exogenous glucose oxidation compared to total glucose oxidation could also be an indirect consequence of the delayed appearance of the ^13^C label in ^13^CO_2_, a known phenomenon due to label retention in the bicarbonate pool in human studies^40,41^ and in agreement with the half-life of CO_2_ of about 15 min in mice^42^. In humans, label sequestration in *de novo* synthesized glucose, glutamine, and glutamate in fatty acid tracer studies likely pertains also to glucose tracers^43^, although this is yet to be investigated in mice. Together with the unrealistically high ^13^C label recoveries we obtained after applying a human dietary acetate recovery factor (dARF) to the situation in mice, our data illustrates the need for a species-specific correction factor.

Integrating InCa with ^13^CO_2_ and ^12^CO_2_ sensors will improve the interpretation of mouse metabolic studies and provide crucial quantitative data. In addition, it will allow a wide variety of specific substrates to be studied, including metabolic substrates with highly variable or prolonged oxidation kinetics. Substrate-specificity could be further aided by the inclusion of other gas sensors. Moreover, a similar sensor technology would obviate some experimental challenges that remain in metabolic research with stable isotopes in humans, like the necessity to interrupt InCa sessions to obtain separate samples for ^13^CO_2_ analysis.

In conclusion, the analysis of ^13^CO_2_ enrichment coupled to conventional InCa is a powerful and targeted tool to quantify the kinetics of exogenous substrate oxidation. We have incorporated ^13^CO_2_ and ^12^CO_2_ sensors into a commercial InCa system for use in the mouse, a human-relevant model organism, and demonstrated its value to study fuel use strategies in physiological conditions, non-invasively, and continuously over long experimental periods.

## Methods

### Integration of ^13^CO_2_ and ^12^CO_2_ sensors into the indirect calorimetry (InCa) system

An Infrared Analyser Module URAS26 for separate analysis of ^13^CO_2_ and ^12^CO_2_ by nondispersive infrared absorption (ABB Automation, Frankfurt am Main, Germany) was incorporated into a 12-cage PhenoMaster InCa system (TSE Systems, Bad Homburg, Germany) in a closed circuit in series, upstream of the standard Siemens High-Speed Sensor Unit containing the standard O_2_ and total CO_2_ analysers (Fig. 1a). The integration of a methane (CH_4_; ABB Automation) and a hydrogen (H_2_; Honeywell Analytics, Hegnau, Switzerland) analyser into our InCa system has been reported previously^16^. The ^12^CO_2_ sensor has a range of 0–6000 ppm which is appropriate in relation to total CO_2_ exchange in mice, since total CO_2_ ambient levels normally lie around 440 ppm and can raise up to about 5000 ppm for single-housed adult mice (based on previous observations in our laboratory). The ^13^CO_2_ sensor has a measuring range of 0–150 ppm suitable to measure natural ^13^CO_2_ concentrations, which are estimated to be 5.5 ppm in ambient air and 55 ppm in mouse cages. Calibration of the equipment was done routinely with three gas mixtures (Linde Gas Benelux, Dieren, The Netherlands): *zero* (20.947% O_2_, in N_2_, no other constituents), *span 1* (98.8 ppm H_2_, in synthetic air), and *span 2* (0.521% total CO_2_, 450 ppm CH_4_, in N_2_). The zero calibration point was performed by flushing the *zero* gas mixture through the system for 10 min and assigning ADC signals their corresponding gas concentration values. The same procedure was repeated for the span calibration points using gas mixtures *span 1* and *span 2*. The ^12^CO_2_ and ^13^CO_2_ span calibration points were set to 5153 and 57 ppm, respectively, based on the natural enrichment of atmospheric CO_2_ (1.1 atom%)^3^. Cross-sensitivity between the ^12^CO_2_ and ^13^CO_2_ analysers is negligible. This calibration routine was performed before each experiment, with each experiment lasting for no more than a week, according to the stability of the ^12^CO_2_ and ^13^CO_2_ zero and span points of < 1% drift per week reported by the manufacturer. Raw data was acquired with a customised version of PhenoMaster software v.5.8.0 (TSE Systems), including ^12^CO_2_ and ^13^CO_2_ concentrations in ppm. Delta ppm values were obtained by subtracting reference cage values from mouse cage values at each time point, and these values were used for further calculations. Other operational settings and procedures have been described previously^29^. The overall performance of the newly extended system was first tested by measuring all gas concentrations over 5 d using empty cages.

### Composition of experimental diets

The highly digestible-starch diet (HDD) and the lowly digestible-starch diet (LDD) contained 20, 55, and 25 energy% protein, carbohydrate, and fat, respectively, and fulfilled the nutritional requirements for rodents according to AIN-93^44^. The starches in HDD and LDD (569 g kg^–1^ diet; Cargill, Sas van Gent, The Netherlands) were incorporated by Research Diet Services (Wijk bij Duurstede, The Netherlands) for the preparation of the pelleted diets. The high fat diet (HFD) contained 20, 40, and 40 energy% protein, carbohydrate, and fat, respectively. The exact composition of the experimental diets has been described in more detail elsewhere^31^.

### Elemental analysis isotope ratio mass spectrometry (EA-IRMS)

The ^13^C enrichments of the chow diet and the HDD and LDD were measured by EA-IRMS as published^45^. Briefly, pulverized samples were combusted at 1020 °C in the presence of oxygen to convert carbon into CO_2_, followed by separation for measurement of the ^13^C/^12^C ratio by EA-IRMS.

### Mouse experiments

The experiments were approved by the Animal Experiment Committee of Wageningen University DEC2014085 and CCD/IvD 2017.w-0024.003, and performed in accordance with the European Union (EU) directives 86/609/EEC and 2010/63/EU, respectively. All mice (C57BL/6JRccHsd, Envigo, Horst, The Netherlands) were individually housed in Makrolon II cages with wood chips and enriched with wood shavings, at 23 ± 1 °C, 50 ± 5% humidity, on a 12 h light/dark cycle. Unless otherwise indicated, mice had *ad libitum* access to food and water.

Two mouse studies were conducted. The first study aimed to validate the newly incorporated ^13^CO_2_ and ^12^CO_2_ sensors using mice fed diets of variable natural ^13^C enrichment. Ten-month old female mice (*n* = 12) raised on a chow diet (AM-II, AB Diets, Woerden, The Netherlands), with wheat as main ingredient and no declared content of C4 plant ingredients (1.078 atom% ^13^C, EA-IRMS, see above), were weighed and acclimatised to the InCa environment for 48 h. Mice were then switched to one of two maize-based semi-purified diets with 57% w/w starch: HDD (1.085 atom% ^13^C, EA-IRMS, see above) or LDD (1.085 atom% ^13^C, EA-IRMS). The allocation of HDD and LDD was randomised and known to the experimenter, and the body weight (BW) of these groups was similar. Air measurements continued for another 4.5 d. Bedding volume was limited to approximately 200 ml during InCa measurements to facilitate detection of voluntary locomotion by infrared beam breaks in the horizontal plane. All gas concentrations were measured continuously. Other data obtained from the animals in this experiment (H_2_ production and gut microbiota composition) has been reported previously^16^.

The second study aimed at quantifying oxidation of ^13^C-labelled exogenous metabolic substrates in diet-induced obesity. Mice on a chow diet (Teklad Global Diet 2920, Envigo) were time-mated and their offspring cross-fostered within 24–48 h after birth. Female offspring (*n* = 48) were weaned at the end of postnatal week (PW) 3, stratified according to BW, and assigned to either HDD or LDD for 3 weeks; the experimenter was not blinded to these dietary treatments. Mice were then switched to a wheat-based HFD in PW 7 and continued on this diet until PW 15. Mice originally on HDD and LDD were initially treated as two experimental groups and, per group, were re-stratified by BW in PW 13 (prior to InCa and refeeding challenges in PW 14–15, see below) and again at the end of PW 15 before sacrifice in the fasted or postprandial condition (see below), to ensure the distribution of BW was similar across subgroups receiving the two differently labelled liquid mixed meals (*n* = 24 for either ^13^C glucose or ^13^C palmitate) and sacrificed in the two metabolic states (*n* = 24 fasted or postprandial). BW and food intake (FI) were determined weekly. Body composition (BC; EchoMRI 100 V, EchoMedical Systems, Houston, Texas, USA) was determined weekly (PW 4–6) or biweekly (PW 7–15), and directly before and after InCa runs. The RER response to refeeding with a liquid mixed meal, circulating fasting and postprandial glucose and insulin levels, and 24 h EE (Supplementary Table S1 and Supplementary Fig. S2) were not different between the mice originally on HDD or LDD. These metabolically very similar mice were then pooled into a group of in total 48 mice with a widely different fat mass (FM), and this combined group was used to investigate the metabolic response to the exogenous substrates of the liquid mixed meals and its correlation with FM.

### InCa and refeeding challenge tests with liquid mixed meals

Individually housed mice (in batches of 12 per InCa run) were acclimatised to the InCa environment for approximately 24 h. The following 24 h period was used for measurements of daily EE, RER, locomotor activity, and food and water intake. Sampling frequency for these basal gas measurements was every 20 min. On the third day, 6 mice per batch, to facilitate a higher gas sampling frequency, were restricted to 1.1 g of food (HFD) 1 h before the dark phase (DP; ZT = 11). The remaining 6 mice kept *ad libitum* access to food and water inside the InCa system. Twenty-four hours later (ZT = 11), the 6 cages with mice receiving restricted food were continuously measured at sample interval of 11 min, and these mice received a ^13^C-labelled liquid mixed meal by oral gavage (0.4 ml per mouse, see below). The mice were continuously monitored in the extended InCa system, including ^13^CO_2_ and ^12^CO_2_ measurements, until the onset of the following light phase (LP; ZT = 0) for a total of 13 h upon ingestion of the liquid mixed meal, after which they regained *ad libitum* access to the HFD. On the fourth day, the same procedure (*i.e*. measurement following food restriction and a subsequent ^13^C-labelled liquid mixed meal) was repeated with the remaining 6 mice per batch in the InCa system. All of the mice received the same dose of liquid mixed meal. The meal contained a mixture of glucose and fat, representing 16.3 ± 1.1% (mean ± SD) of daily energy expenditure, and contained either a ^13^C glucose or a ^13^C palmitate tracer (Table 2), allowing determination of glucose or palmitic acid oxidation specifically.

**Table 2:** Composition of the liquid mixed meals.

	^13^C Glucose	^13^C Palmitate
Unlabelled glucose (mg)	1358.4 ± 0.7	1401.3 ± 1.1
U-^13^C Glucose (mg)	43.1 ± 0.4	0
Palm olein (mg)	977.5 ± 2.3	974.3 ± 2.0
Unlabelled sodium palmitate (mg)	23.9 ± 0.2	0
U-^13^C Potassium palmitate (mg)	0	26.7 ± 0.2
Soy lecithin (mg)	8.1 ± 0.9	7.9 ± 0.6
Water (mg)	1501.7 ± 2.8	1501.0 ± 1.1
Density (g ml^–1^)	1.12 ± 0.01	1.11 ± 0.02
^13^C content (µmol)^a^	157.2 ± 2.8	155.1 ± 2.5
Energy (kJ)^a^	7.19 ± 0.09	7.16 ± 0.12
Carbohydrate (energy%)	36.0 ± 0.1	36.0 ± 0.1
Fat (energy%)	64.0 ± 0.1	63.9 ± 0.1

^a^Amount per dose of 0.4 ml of liquid mixed meal given by oral gavage. Data is presented as mean ± SD.

### Design and preparation of liquid mixed meals

The liquid mixed meals were based on a recently developed drink used to measure the metabolic response to refeeding and health in humans^46^. However, as the focus was exclusively on exogenous glucose and fat oxidation, and therefore included corresponding metabolic tracers (^13^C glucose or ^13^C palmitate), we omitted protein from the formulation. Palmitate was chosen as a fat tracer instead of labelled triglycerides or a mixture of fatty acids to circumvent possible fatty-acid-specific differences in absorption and oxidation. Sodium palmitate (Sigma-Aldrich, Missouri, State, USA) or D-glucose (Merck, Darmstadt, Germany) were partly replaced by either uniformly (U) ^13^C-labelled potassium palmitate (98.8 atom%, 98% chemical purity; IsoLife, Wageningen, The Netherlands) or U-^13^C D-glucose (99 atom%, 98.8% chemical purity; IsoLife), respectively, and mixed with soy lecithin (Emultop IP, Cargill, Hamburg, Germany) and ultrapure water. This mixture was then vortexed and microwaved until no visible lumps remained. Palm olein (Remia, Den Dolder, The Netherlands) was added and the aqueous and oily phases were integrated by vortexing and sonication until a homogenous emulsion was obtained. Fresh preparations were made 2 h before administration to the animals and remained stable. The overall composition of the labelled glucose and labelled palmitate liquid mixed meals is shown in Table 2.

### Sacrifice in the fasted or postprandial state

Mice were food restricted (1.1 g HFD) for 16 h, starting at ZT = 11. Half of the mice was sacrificed in the post-absorptive state, and the other half was administered the liquid mixed meal with ^13^C glucose by oral gavage and sacrificed after 45 min (postprandial state) by decapitation. Trunk blood was collected in MiniCollect serum tubes (Greiner Bio-One, Alphen aan de Rijn, The Netherlands). Serum was separated by centrifugation at 4 °C for 10 min at 3000 × *g*, aliquoted, and stored at −80 °C. Glucose was measured in whole blood with a Freestyle glucose meter (Abbott Diabetes Care, Hoofddorp, The Netherlands) directly after sacrifice.

### Serum insulin measurements

Fasting and postprandial serum insulin concentrations from animals sacrificed in PW 15 were determined with an Ultra-Sensitive Mouse Insulin ELISA Kit (ChrystalChem, Elk Grove Village, Illinois, USA) following the manufacturer’s instructions. Samples were measured in duplicate.

### Calculation of refeeding response (∆RER), and total and exogenous substrate oxidation

The metabolic refeeding response (∆RER) was determined per individual animal as the change in RER from the fasting post-absorptive state (baseline over 1 h) to postprandial 44 min after administering the liquid mixed meal, based on the median time when all mice achieved a postprandial RER peak. Total levels of glucose and fatty acid oxidation were calculated from VO_2_ and VCO_2_ obtained with the Siemens High-Speed Sensor Unit, using Péronnet & Massicotte’s table of non-protein RER^17^ and Weir’s equation of EE^47^, as follows. Individual values for glucose and fatty acid oxidation (as % of EE) were interpolated from the original table of Péronnet & Massicotte, ranging from RER 0.7036 to 0.996. These interpolated values of glucose or fatty acid utilisation (% of EE) and the EE data [also obtained from the TSE system, based on Weir’s equation: EE = (3.941 × VO_2_) + (1.106 × VCO_2_); kJ min^−1^], together with the energy equivalents of glucose and fatty acids (16.18 kJ g^−1^ and 40.76 kJ g^−1^, respectively^17^), were then used to calculate the rates of total glucose oxidation (TGO) and total fatty acid oxidation (TFO) in mg min^−1^, according to the following equations:

1 $$TGO=\frac{EE\times  \% E{E}_{GO}}{16.18}$$

2 $$TFO=\frac{EE\times  \% E{E}_{FO}}{40.76}$$

Rates of exogenous substrate oxidation (ESO; *i.e*. oxidation of ingested glucose and palmitate in the liquid mixed meal based on the ^13^C tracers) were calculated using the following two equations:

3 $$at \% {}^{13}C{O}_{2}=\frac{{}^{13}C{O}_{2}}{{}^{13}C{O}_{2}+{}^{12}C{O}_{2}}\times 100$$

4 $$ESO(mg\times mi{n}^{-1})=\frac{at \% {}^{13}C{O}_{{2}_{(t)}}-at \% {}^{13}C{O}_{{2}_{(t0)}}}{at \% {}^{13}C_{S}-at \% {}^{13}C{O}_{{2}_{(t0)}}}\times \frac{VC{O}_{{2}_{(t)}}\times M{W}_{tracee}}{22.2966\times {C}_{tracer}}$$

In equations (3) and (4), at%^13^CO_2_ is the ^13^C enrichment in expired CO_2_ in atom% calculated from gas concentrations (delta ppm). In equation (4), time t_0_ represents the baseline measurement over 1 h before administration of the liquid mixed meals and t represents any subsequent time point. The calculated ^13^C enrichment of the whole substrate pool ingested (either unlabelled glucose plus ^13^C glucose, or unlabelled palmitate plus ^13^C palmitate) is represented by at %^13^C_s_, assuming a natural terrestrial ^13^C enrichment of 1 atom% (ref.^4^, 100% chemical purity, and following the fatty acid composition of palm olein from literature^48^. VCO_2_ is the production rate of CO_2_ obtained using the summed concentrations of ^13^CO_2_ and ^12^CO_2_ measured by the URAS26 module multiplied by the air flow (constant). The molecular weight of the tracee (MW_tracee_, glucose or palmitic acid) and the volume occupied by 1 mol of CO_2_ in STPD (22.2966 l) are based on Péronnet & Massicotte^17^. The number of labelled carbons per mol of tracer (C_tracer_) is 6 for U-^13^C glucose and 16 for U-^13^C palmitate. Of note, exogenous fat oxidation (EFO) represents only the oxidation of palmitic acid ingested (both labelled and unlabelled), thus oxidation of other fatty acids in the liquid mixed meal (mainly oleic acid and linoleic acid) is not accounted for.

Additionally, a dietary acetate recovery factor (dARF) was implemented in our calculations giving rise to an alternative ESO calculation, equation (5). The dARF is a factor suggested to be used to correct for ^13^C sequestration based on studies in normal weight and obese humans^19,49^, but has not been validated in mice. In detail, the animal with the highest FM and the animal with the lowest FM were assigned the dARF of obese (0.453) and normal weight humans (0.506), respectively, and the dARF of the remaining animals was interpolated by linear regression and applied to equation (4):

5 $$ESO(mg\times mi{n}^{-1})=\frac{at \% {}^{13}C{O}_{{2}_{(t)}}-at \% {}^{13}C{O}_{{2}_{(t0)}}}{at \% {}^{13}C_{S}-at \% {}^{13}C{O}_{{2}_{(t0)}}}\times \frac{VC{O}_{{2}_{(t)}}\times M{W}_{tracee}}{22.2966\times {C}_{tracer}\times dARF}$$

### Statistical analysis

Each individual mouse was considered an experimental unit. Normal distribution of the data was tested with the D’Agostino and Pearson omnibus test; non-normally distributed data were log-transformed and retested for normality. The difference in natural ^13^C enrichment on wheat- *vs* maize-based diets was analysed with the Kruskal-Wallis test with Dunn’s multiple comparison test. Correlations were performed by Pearson correlation (normally-distributed data) or Spearman correlation (non-normally distributed data). Technical errors occurred in oral gavage in two animals, therefore this data was not included for analyses of postprandial metabolic outcomes. All statistical analyses and data visualisation were performed in Prism v.5.04 (GraphPad, San Diego, California, USA). Data is presented as mean ± SD (normally distributed data) or median and range (non-normally distributed data), and statistical significance was set at *P* < 0.05.

## Supplementary information

Supplementary information for: Extended indirect calorimetry with isotopic CO_2_ sensors for prolonged and continuous quantification of exogenous vs. total substrate oxidation in mice

## Data Availability

All data generated and analysed during this study is included in this published article and its Supplementary Information files.
