# Supplementary material for: Extended indirect calorimetry with isotopic CO2 sensors for prolonged and continuous quantification of exogenous vs. total substrate oxidation in mice
Source: Sci Rep. 2019 Aug 8;9:11507. doi: 10.1038/s41598-019-47977-w (PMC6687832; doi:10.1038/s41598-019-47977-w)
Supplement: Supplementary file 1 — Supplementary information for: Extended indirect calorimetry with isotopic CO2 sensors for prolonged and continuous quantification of exogenous vs. total substrate oxidation in mice [file 41598_2019_47977_MOESM1_ESM.pdf]

Supplementary information for:

Extended indirect calorimetry with isotopic CO_2 sensors for prolonged and continuous quantification of exogenous *vs.* total substrate oxidation in mice

José MS Fernández-Calleja[1], Lianne MS Bouwman[1], Hans JM Swarts[1], Annemarie Oosting[2], Jaap Keijer[1], Evert M van Schothorst[1*]

[1]Human and Animal Physiology, Wageningen University, De Elst 1, Wageningen 6708 WD, The Netherlands

[2]Danone Nutricia Research, Uppsalalaan 12, Utrecht 3584 CT, The Netherlands

Supplementary Table S1 and Supplementary Figures S1 and S2

Supplementary Table S1. Metabolic characteristics of the female C57BL/6JRccHsd mice fed HDD and LDD from postnatal week (PW) 4 to PW 6, and HFD from PW 7 to PW 15 (second mouse study).

Parameter	PW 6			PW 15		
	HDD	LDD	*P*-value	HDD	LDD	*P*-value
BW (g)	17.02 (15.40, 19.28)	17.42 (15.35, 18.49)	0.0266	30.69 ± 3.00	32.44 ± 3.63	0.0744
FM (g)	1.51 (0.76, 2.83)	1.47 (0.82, 2.65)	0.8902	9.38 ± 2.47	11.04 ± 3.06	0.0442
LM (g)	14.56 ± 0.70	14.83 ± 0.67	0.1794	19.87 ± 0.91	19.90 ± 0.69	0.8744
FM gain (g)[1]	0.63 (0.02, 1.96)	0.66 (-0.07, 1.80)	0.9425	7.85 ± 2.29	9.48 ± 2.96	0.0381
dEI (MJ)[1]	0.88 (0.73, 1.05)	0.97 (0.86, 1.04)	< 0.0001	3.67 ± 0.30	3.71 ± 0.25	0.5839
24 h EE (kJ d^{-1})[2]	ND	ND	-	43.93 ± 2.91	44.55 ± 2.99	0.4745
24 h RER[3]	ND	ND	-	0.82 ± 0.04	0.80 ± 0.03	0.0698
ΔRER (RER_{44} – RER_0)[2]	ND	ND	-	0.17 (0.10, 0.19)	0.16 (0.11, 0.20)	0.4436
Fasting glucose (mmol l^{-1})	ND	ND	-	5.8 (4.9, 7.2)	6.0 (5.2, 7.1)	0.5245
Fasting insulin (ng ml^{-1})	ND	ND	-	2.72 (0.85, 8.95)	2.31 (1.06, 3.45)	0.6049
Postprandial glucose (mmol l^{-1})	ND	ND	-	7.1 ± 1.3	6.8 ± 1.7	0.6811
Postprandial insulin (ng ml^{-1})	ND	ND	-	4.31 ± 2.85	4.48 ± 1.42	0.8622

Supplementary Table S1. Metabolic characteristics of the female C57BL/6JRccHsd mice fed HDD and LDD from postnatal week (PW) 4 to PW 6, and HFD from PW 7 to PW 15 (second mouse study). [1]Measured from PW 4 to PW 6 (post-weaning period on HDD or LDD), or from PW 7 to PW 15 (HFD-feeding). [2]Measured in PW 14-15. HFD, high-fat diet; HDD, highly-digestible starch diet; LDD, lowly-digestible starch diet; BW, body weight; dEI, digestible energy intake; FM, fat mass; LM, lean mass; EE, energy expenditure; ND, not determined; RER, respiratory exchange ratio (mean of 24 h). Data was analysed separately for PW 6 and PW 15 time points using a Student’s *t*-test (normally

distributed data) or a Mann Whitney test (non-normally distributed data). Data is presented as median and range (BW PW 6, FM PW 6, FM gain PW 6, dEI PW 6, ΔRER, fasting blood glucose, fasting blood insulin) or otherwise as mean ± SD, n = 24 per group (except ΔRER, HDD: n = 24, LDD: n = 23; fasting glucose and insulin, n = 12 per group; and postprandial glucose and insulin, HDD: n = 11, LDD: n = 12)

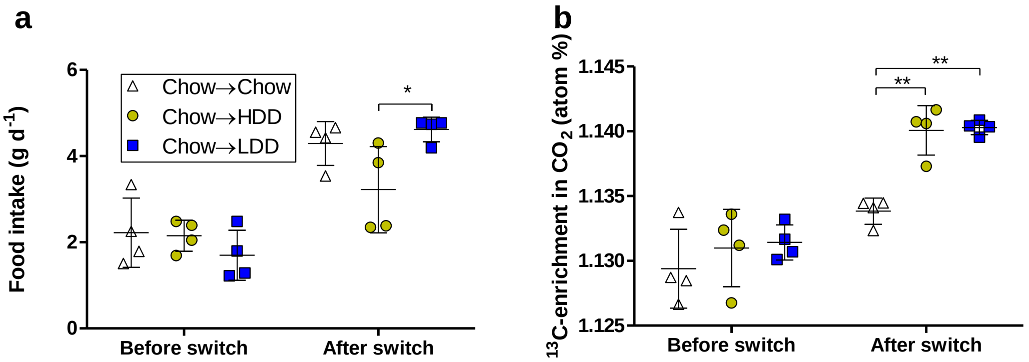

Source of Variation	% of total variation	P value
Interaction	9.35	0.0150
Switch	66.48	< 0.0001
Diet	4.05	0.3203
Subjects (matching)	14.0595	0.1130

Source of Variation	% of total variation	P value
Interaction	5.23	0.0623
Switch	63.61	< 0.0001
Diet	16.93	0.0062
Subjects (matching)	8.0954	0.3430

Supplementary Figure S1. Changes in food intake and $^{13}CO_2$ enrichment in mice fed chow and then switched HDD or LDD, or remaining on chow. Chow-fed mice (*n* = 12) were adapted to indirect calorimetry cages for 2 d, after which mice were allowed a restricted amount (1.1 g) of chow 1 h before the onset of the dark phase. At the end of the following light phase, mice were re-fed with a restricted amount (1.1 g) of chow, or HDD or LDD (*n* = 4). Before the following dark phase mice received *ad libitum* access to the same diet they were allocated the day before, and measurements continued for 5.5 d (see Fernández-Calleja *et al.*[1] for details). (**a**) 24 h cumulative food intake and (**b**) 24 h mean ^{13}C enrichment immediately before food restriction and the day after *ad libitum* re-feeding. Tables show statistical output of repeated-measures 2-way analysis of variance. *$P < 0.05$, **$P < 0.01$, Bonferroni multiple comparisons. Data is presented as mean ± SD. HDD, highly digestible starch-diet; LDD, lowly digestible starch-diet.

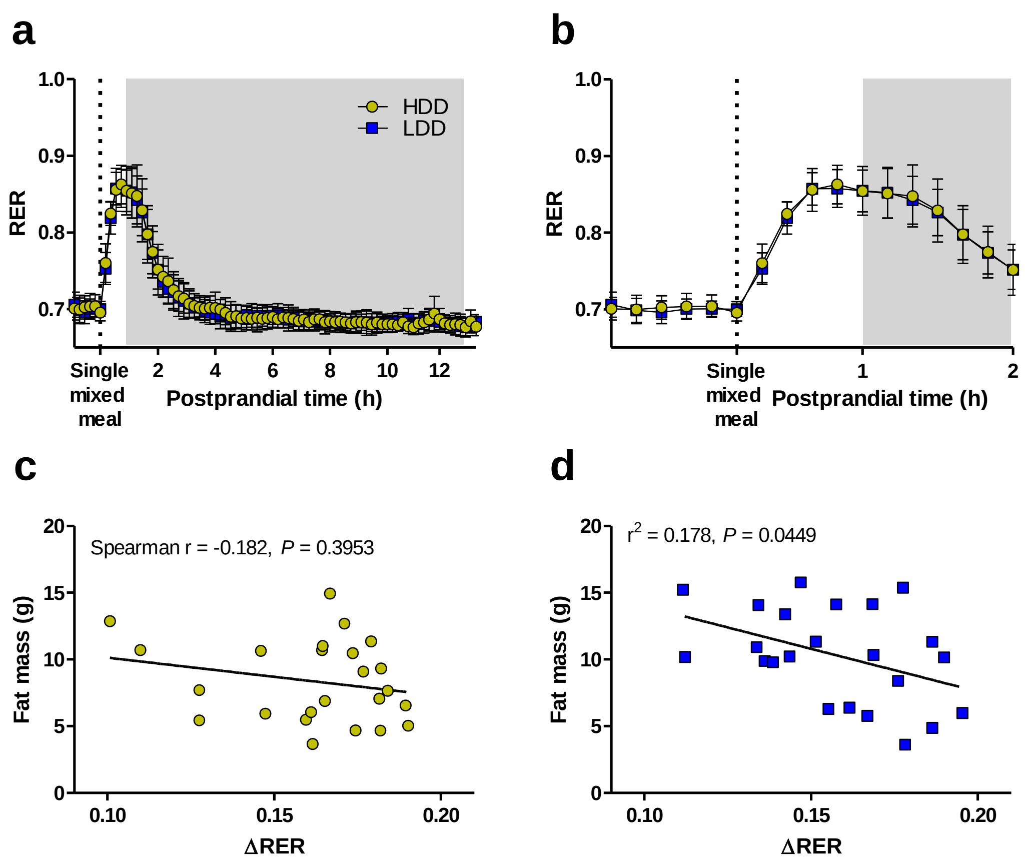

Supplementary Figure S2. Refeeding metabolic response to a liquid mixed meal and its relation to body fat mass (FM) in mice fed a HFD for nine weeks (second mouse study). (**a**) Respiratory exchange ratio (RER) was measured in PW 14-15 after gavage of a single liquid mixed meal containing 36 energy% glucose and 64 energy% fat in mice fed the HDD (n = 24) or the LDD (n = 23) during early life and subsequently fed a HFD for nine weeks. Data was analysed with repeated-measures 2-way analysis of variance to directly test the hypothesis that RER response is influenced by the post-weaning diet. There were no significant effects of the post-weaning diet or its interaction with time. Data is presented as mean ± SD. Shaded areas represent the dark phase. (**b**) Data from panel (**a**), emphasizing the early postprandial 2 h period. Correlation analysis between FM and the re-feeding response (ΔRER), measured as the increase from baseline RER to RER at 44 min postprandial, in mice originally fed HDD (**c**) or LDD (**d**) during early post-weaning. HDD, highly digestible starch-diet; LDD, lowly digestible starch-diet.

Reference

1. Fernández-Calleja, J. M. S. *et al*. Non-invasive continuous real-time *in vivo* analysis of microbial hydrogen production shows adaptation to fermentable carbohydrates in mice. Sci. Rep. **8**, 15351, doi:10.1038/s41598-018-33619-0 (2018).
